# Identification of Prediabetes Discussions in Unstructured Clinical Documentation: Validation of a Natural Language Processing Algorithm

**DOI:** 10.2196/29803

**Published:** 2022-02-24

**Authors:** Jessica L Schwartz, Eva Tseng, Nisa M Maruthur, Masoud Rouhizadeh

**Affiliations:** 1 Division of General Internal Medicine Johns Hopkins School of Medicine Baltimore, MD United States; 2 Division of Hospital Medicine Johns Hopkins Hospital Baltimore, MD United States; 3 Welch Center for Prevention, Epidemiology, & Clinical Research Johns Hopkins University Baltimore, MD United States; 4 Department of Epidemiology Johns Hopkins University Bloomberg School of Public Health Baltimore, MD United States; 5 Department of Pharmaceutical Outcomes and Policy University of Florida College of Pharmacy Gainesville, FL United States; 6 Division of Biomedical Informatics and Data Science Johns Hopkins University School of Medicine Baltimore, MD United States

**Keywords:** prediabetes, prediabetes discussions, prediabetes management, chronic disease management, physician-patient communication, natural language processing, machine learning

## Abstract

**Background:**

Prediabetes affects 1 in 3 US adults. Most are not receiving evidence-based interventions, so understanding how providers discuss prediabetes with patients will inform how to improve their care.

**Objective:**

This study aimed to develop a natural language processing (NLP) algorithm using machine learning techniques to identify discussions of prediabetes in narrative documentation.

**Methods:**

We developed and applied a keyword search strategy to identify discussions of prediabetes in clinical documentation for patients with prediabetes. We manually reviewed matching notes to determine which represented actual prediabetes discussions. We applied 7 machine learning models against our manual annotation.

**Results:**

Machine learning classifiers were able to achieve classification results that were close to human performance with up to 98% precision and recall to identify prediabetes discussions in clinical documentation.

**Conclusions:**

We demonstrated that prediabetes discussions can be accurately identified using an NLP algorithm. This approach can be used to understand and identify prediabetes management practices in primary care, thereby informing interventions to improve guideline-concordant care.

## Introduction

Prediabetes affects 88 million US adults [1,2], and evidence-based interventions focusing on lifestyle modification can prevent type 2 diabetes [3-12]. In particular, the Diabetes Prevention Program is an effective lifestyle intervention that decreases diabetes incidence, with the most recent data showing a 27% risk reduction compared with the placebo arm over 15 years of follow up [5]. Unfortunately, up to 89% of patients do not know they have prediabetes [13,14], and many patients are unaware of interventions to decrease their risk of diabetes—relying on their primary care providers (PCPs) to initiate discussions about diabetes prevention, including the importance of lifestyle changes [8,9]. However, survey data demonstrate that many providers feel that they lack the resources to effectively implement evidence-based prediabetes treatment [8,9]. Focused primary care interventions to support decision-making and education may be able to improve diagnosis of prediabetes and delivery of guideline-concordant care.

Rigorous quality improvement interventions require evaluation using measurement before and after implementation of a project to determine whether there is a demonstrable change in target outcomes. Unfortunately, it is difficult to identify changes and improvement in prediabetes management through structured data alone. Relying on diagnosis codes is insufficient; one study showed that only 13% of patients with prediabetes had an International Classification of Diseases (ICD)-9 diagnosis of prediabetes or hyperglycemia [14]. Although labs, orders, and referrals provide some insight, this information lacks detail about management, particularly lifestyle counseling, which is better captured in narrative documentation. This content is not easily queried and requires innovative research methods to accurately reflect delivery of prediabetes care.

Prior studies have shown that natural language processing (NLP) can be used to diagnose chronic conditions, like diabetes, but few focus on disease management [15]. Similarly, NLP studies in prediabetes have primarily focused on disease detection, screening, and predictive modeling, with no studies applying machine learning (ML) techniques to determine how prediabetes is managed [16-27]. Our goal was to develop a method to identify when providers discuss prediabetes management and treatment, which could later be used to determine if care delivered meets evidence-based guidelines and compare outcomes before and after an intervention. Therefore, we developed and validated NLP pipelines to identify primary care discussions about prediabetes in clinical documentation.

## Methods

### Population and Ethics Approval

We identified patients with prediabetes who had an internal medicine primary care visit within an academic center with multiple ambulatory locations in Maryland and Washington, DC. Eligible patients were adults (≥18 years old) covered by 1 of 3 major insurers who completed an in-person visit and had a hemoglobin A_1c_ (HbA_1c_) level between 5.7% and 6.4% between July 1, 2016 and December 31, 2018. Patients with diabetes (any type) based on billing codes or documentation in the problem list or past medical history were excluded. Data cleaning and analyses were performed using Stata 15. This study was approved by the Johns Hopkins Institutional Review Board (IRB00196984).

### Keyword Search Refinement (Phase 1)

Based on clinical experience, we developed a list of keywords used to describe “prediabetes” (Table S1 in Multimedia Appendix 1). We identified visit notes containing these keywords using Python string matching and dictionary look-up, accounting for variations like spelling errors and morphological differences. We extracted a ±25-word concordance window (“note snippet”) for each match to provide textual context. Multiple snippets could come from the same note if multiple matching keywords were present.

We selected 2 ambulatory clinics from our overall population. Of 315 patients meeting inclusion criteria, 40.6% (128/315) had at least one matching keyword during the study period. These patients had a total of 637 keyword matches across 324 encounters with 25 providers. We conducted manual annotation to determine which of the 637 note snippets represented true clinical discussions of prediabetes (yes or no). Outpatient provider documentation typically includes chief complaint, history of present illness, medical and family history, objective data including physical exam, and an assessment and plan. We considered use of a section identification pipeline to exclude specific sections of the notes (eg, past medical history) in which keywords would not represent prediabetes discussions. However, section identification pipelines are less generalizable, and the providers in our sample did not use standardized templates, making section boundaries difficult to define [28]. Instead, note snippets were designated “no” during manual review if the keyword was only present in past medical history, a list of diagnoses outside of the assessment and plan, family history, or the description of a lab result.

We double-reviewed a random sample of 200 note snippets. Interrater reliability (IRR) was 95%. Discrepancies between annotators were resolved via consensus to refine the definition of “prediabetes discussion.” We then manually reviewed patient records for 35.3% (66/187) of charts without a keyword match to identify false negatives. We reviewed all notes written by the patient’s PCP within the inclusion timeframe, and 9% (6/66) of patients had prediabetes discussions that were not captured. We added 3 keywords (“dysglycemia,” “hyperglycemia,” and “pre diabetes”) to the lexicon (Table S1 in Multimedia Appendix 1).

### Training Set (Phase 2)

**Figure 1:**
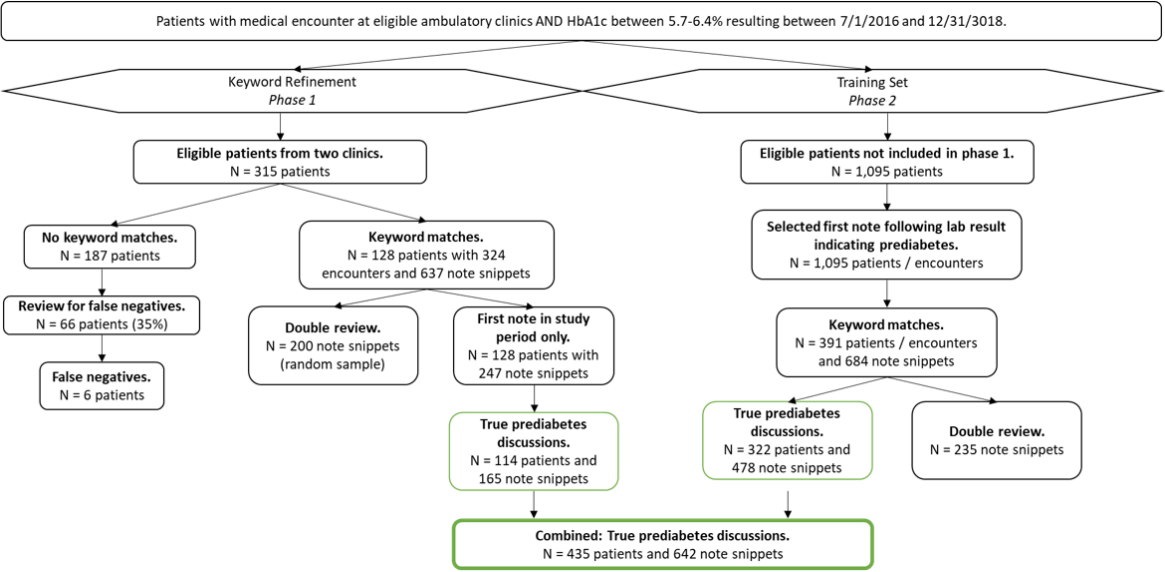
Diagram depicting selection and review during keyword search refinement (Phase 1) and training set development (Phase 2). Eligible patients were adults (≥18 years old) covered by 1 of 3 major insurers who completed an in-person visit at a Johns Hopkins clinic and had an HbA_1c_ level between 5.7% and 6.4% (39-46 mmol/mol) between July 1, 2016 and December 31, 2018. Note, double review indicates that 2 providers reviewed the keyword matches to identify whether the surrounding text represented a true prediabetes discussion.

We developed a training set to test our prediabetes lexicon against patients from clinics not included in phase 1 (Figure 1). We included a single note per patient (n=1095), choosing the first encounter after the HbA_1c_ result that met inclusion criteria. We applied the finalized keyword search, which resulted in 684 matches for 381 patients seen by 73 providers. We abstracted the 684 note snippets and annotated the notes using a similar process as above. We double-reviewed 34% of the note snippets with an IRR of 97% for manual annotation, resolving to 100% agreement upon review. We combined these results with note snippets from phase 1. To avoid overselection of a single patient or provider, we included note snippets from 1 encounter per patient for a total of 930 note snippets written by 96 unique providers.

### Rule-Based System

Rule-based systems are frequently used for clinical concept extraction and text classification systems because of their ease of implementation and minimal computational requirements. To establish a strong baseline, we tested the feasibility of identifying prediabetes discussions with a rule-based classification scheme. Using the spaCy EntityRuler module [29], we created 42 expert-developed patterns that, if present, would represent prediabetes discussions. The spaCy EntityRuler module facilitates various pattern, keyword, and regular expression searching and matching and allows us to account for morphological variations (eg, singular vs plural forms, conjunctions), as well as substitutions of different prepositions (eg, about vs for) and synonyms (eg, prediabetes, impaired fasting glucose). Table S2 in Multimedia Appendix 1 provides our expert-developed patterns for this rule-based system. We randomly sampled 90% of the note snippets to develop and revise the rule-based system and evaluated the system on the remaining 10%.

### Machine Learning

#### Feature Selection

Note snippets from the training set were stemmed using the Porter stemmer, and common stop words were removed using the Natural Language Toolkit (NLTK) stop word list [30]. We used the Python scikit-learn library [31] to extract word ngram sequences (1-5 grams), weighted by term frequency-inverse document frequency (TF-IDF) [32]. We applied logistic regression with L1 regularization [33] to reduce the dimensionality of the feature vectors.

#### Computational Environment

Deep learning and ML experiments were conducted on the Johns Hopkins University (JHU) Precision Medicine Analytics Platform (PMAP), a high-performance, cloud-based, big-data platform to accelerate biomedical discovery and translate discovered knowledge to improve patient-centered care. PMAP pulls data from the Johns Hopkins Medicine electronic health record (EHR) to support processing by ML and NLP technologies. Statistical analysis and manual annotation were done in the JHU Secure Analytic Framework Environment, a virtual desktop that provides JHU investigators with a secure platform for analyzing and sharing sensitive data (including protected health information) with colleagues.

#### Classification

We used the labeled note snippets to train multiple ML classifiers to replicate human annotation for prediabetes discussions. We applied 6 binary classification models: logistic regression [34], linear support vector machines (SVM) [35], stochastic gradient descent (SGD) [36], decision tree [37], random forest [38,39], and Gaussian naïve Bayes (NB) [40]. To reduce overfitting, each model was evaluated using 10-fold cross-validation by training, randomly, on 90% of the data and holding out 10% for testing. All modeling was performed in scikit-learn [31].

We also applied convolutional neural networks (CNNs) for sentence categorization [41], a well-established deep learning method in NLP for text classification [42] using Python spaCy 2.1 implementation [29]. We started with the tokenization of each note snippet and creating an embedding vector of each token using scispaCy large models (~785,000 vocabulary and 600,000 word vectors), pretrained on biomedical and clinical text [43]. Next, to represent the tokens in context, these vectors were encoded into a sentence matrix by computing the vector for each token using a forward pass and a backward pass. After that, a self-attention mechanism was applied to reduce the dimensionality of the sentence matrix representation into a single context vector. Finally, these vectors were average-pooled and used as features in a simple feed-forward network for predicting true discussions of prediabetes. For the CNN model, we used the spaCy 2.2 default network architecture and parameters [44].

For each classification method, we reported on agreement, sensitivity and recall, specificity, positive predictive value and precision, and F measure using manual annotation as the gold standard. To test statistical significance between classification methods, we used MLxtend Python library to perform a 5x2 cross-validation paired *t* test [45]. A *P* value <.05 indicated that we could reject the null hypothesis that both models performed equally to classify prediabetes discussions.

## Results

We identified 1410 patients with prediabetes; 518 (36.74%) had at least one keyword match. Among these patients, 435 (84.0%) had a true discussion about prediabetes in the manually reviewed documents (Figure 1).

The rule-based system was inadequate for replicating human performance, with 72.5% recall and 42.6% specificity (Table 1). ML and CNN classification, however, were close to human performance across all models (Table 1). When comparing conventional classifiers with logistic regression (which had the highest agreement), only linear SVM and NB had similar performance (*P*=.11 and *P*=.15, respectively). CNN outperformed all conventional ML classifiers (logistic regression: *P*=.04; SVM: *P*=.02; SGD: *P*=.002; random forest: *P*=.002; decision tree: *P*=.001; NB: *P*=.03).

**Table 1:** Performance of machine learning methods to approximate manual annotation in identifying prediabetes discussions from primary care note snippets (n=930).

Method		Instances classifier agreed with manual annotation, n (%)	Sensitivity/recall	Specificity	PPV^a^/precision	F measure
**Rule-based system**						
	Expert-developed patterns	588 (63.2)	0.725	0.426	0.737	0.731
**Machine learning**						
	Logistic regression	885 (95.2)	0.966	0.921	0.965	0.965
	Linear support vector machines	878 (94.4)	0.962	0.903	0.957	0.960
	Stochastic gradient descent	858 (92.3)	0.926	0.915	0.96	0.943
	Random forest	863 (92.8)	0.961	0.854	0.937	0.948
	Decision tree	832 (89.5)	0.923	0.83	0.925	0.924
	Gaussian naïve Bayes	883 (95.0)	0.966	0.912	0.96	0.963
	Convolutional neural networks	910 (97.9)	0.984	0.966	0.984	0.984

^a^PPV: positive predictive value.

Manual annotation revealed a variety of linguistic patterns that did and did not represent clinical discussions of prediabetes (Table 2). Most commonly, true discussions were found in the assessment and plan, and those that did not were auto populated from structured fields. ML did result in 5% misclassification based on logistic regression, the best performing conventional classifier; a pattern was not apparent on review of these misclassified note snippets.

**Table 2:** Example text from clinical documentation containing keywords matching the “prediabetes” extraction lexicon, stratified by whether the text represents documentation of a prediabetes discussion.

Location in note			Representative text from note snippets^a^
**Text containing keyword matches representing prediabetes discussions.**			
	Chief complaint	Chief complaint: Patient is a 42 y.o. female here with questions about prediabetes. Patient presents to the visit for an annual physical and reevaluation of HTN^b^ and impaired fasting glucose.	
	History of Present Illness	Has a treadmill but not using regularly. Recent a1c was 6.2 consistent with pre-diabetes.	
	Visit Problem List	Problem List Items Addressed This Visit Asthma Borderline diabetes Essential hypertension Assessment Order Plan 1. Hyperlipidemia ... 7. Impaired fasting glucose 8. Health care maintenance	
	Assessment & Plan	Hyperglycemia Lifestyle modification including diet and exercise discussed. 6. Elevated blood pressure. Pre-diabetes Assessment: recent A1C in good range. Plan: exercise and healthy food changes.	
**Text containing keyword matches not representing prediabetes discussions.**			
	One-liner	Patient with history of HTN, HLD^c^, prediabetes, scleroderma here for routine health assessment.	
	Past Medical History	Past Medical History: Diagnosis Date Asthma 5/14/2008 ... Prediabetes 2/6/2012 Osteoporosis 5/14/2008	
	Problem List	... Hyperlipidemia E78.5 Impaired fasting glucose R73.01 Overweight E66.3 ...	
	Diagnosis list	Diagnoses of Essential hypertension, Osteoporosis, ..., Prediabetes, Asthma, ...	
	Family history	Family History Problem Relation Age of Onset Diabetes Father Prediabetes Paternal Grandfather...	
	Pertinent positive	Diagnosis remains unclear. He has prediabetes. Reports 2-3 months of intermittent palpitations.	
	Pertinent negative	Likely has peripheral neuropathy. Negative RPR^d^, HIV, pre-diabetes.	
	Follow up reasons	Follow up in 1 month for flu shot and prediabetes discussion.	
	Results^e^	For someone without known diabetes, a hemoglobin A_1c_ value between 5.7 % and 6.4 % is consistent with prediabetes and should be confirmed.	
	General guidelines^e^	Type 2 diabetes or prediabetes All men beginning at age 45 and men without symptoms at any age who are overweight or obese and have 1 or more other risk factors.	

^a^Text was modified for length and content to serve as general examples while protecting patient anonymity.

^b^HTN: hypertension.

^c^HLD: hyperlipidemia.

^d^RPR: rapid plasma reagin.

^e^Populated in notes from clinical decision support tools.

## Discussion

### Principal Findings

We utilized NLP and ML techniques to identify prediabetes discussions from unstructured narrative documentation with up to 98% precision and recall. To date, NLP techniques have been used in prediabetes for screening, diagnosis, risk stratification, predictive modeling, and intervention design [16-27,46-50]. To our knowledge, this is the first NLP tool to identify prediabetes discussions. NLP methods have been applied in health care in many ways including in EHR free-text clinical notes to classify disease phenotype, with most studies using simple methods like shallow classifiers or combined with rule-based methods [15,51]. Compared with these studies, our NLP methods are not novel, but our application to disease management distinguishes our study from those that primarily focus on condition identification for chronic diseases [15].

In our study, a simple rule-based system was inadequate to identify prediabetes discussions due to poor specificity. In contrast, all ML methods performed well, with 89% to 98% accuracy. This result demonstrates that prediabetes discussions, despite a variety of documentation styles, can be identified using NLP pipelines. Logistic regression, an efficient conventional classifier with minimal technical dependencies, was statistically outperformed by CNN, a deep learning technique. However, both identified >95% of prediabetes discussions, suggesting that either method could be applied depending on system needs.

Our NLP tool has multiple applications. The simplicity of logistic regression allows for deployment in operational settings, particularly clinical decision support. The tool can also simplify the analytic process before and after a clinical intervention intended to change provider practices. For example, it can isolate discussions about prediabetes, a task that otherwise requires time-consuming manual review. The context of these discussions could then be reviewed to understand the impact of an intervention. This process would strengthen the evaluation of quality improvement programs for prediabetes to promote guideline-concordant care, which includes lifestyle counseling [3-7]. These methods should be replicable to identify conversations about behavioral interventions for other conditions, such as obesity, polysubstance abuse, or tobacco use, that rely heavily on counseling in addition to medication management and referrals.

### Strengths

Our study has several strengths. The keyword refinement stage was rigorous. We validated the initial keyword list against a random sample from 2 ambulatory clinics, ensuring we reviewed a variety of documentation styles. Manual annotation was performed by 2 experts to standardize our definition of “prediabetes discussion,” leading to improvement in IRR scores during training set development. We also identified false negatives and revised our initial keyword list accordingly to ensure capture of prediabetes discussions. Finally, we applied the search criteria developed during keyword refinement to a new set of notes from unique clinics to reduce overfitting. There was a total of 96 different providers included in the 930 unique note snippets, which allowed the model to learn the vocabulary and writing styles of many different clinicians.

### Limitations

Limitations of our study include collection of data from a single health system. However, the clinics included represent urban and suburban sites serving patients of different socioeconomic levels and disease burden, improving generalizability. Providers at other institutions may use different medical terminology, not considered in this study, to describe “prediabetes.” This could limit generalizability outside of the home-trained institution. However, we took several steps to reduce institutional bias, including rigorous keyword refinement and application of the final lexical search to multiple clinics that do not share standardized templates to include many linguistic styles and patterns. We limited our note selection to the first encounter following the abnormal HbA_1c_ result; although this could miss some dialogue about prediabetes, logically these discussions are most likely to occur close to the time of the abnormal result, and this decreased bias in our models. Finally, the note selection process, requiring at least one prediabetes keyword to enter our data set, limited our ability to calculate true recall. We minimized this issue by performing manual review on a subset of the charts that did not enter our data set, to ensure we did not have selection bias in our keyword search. Future studies may consider applying our NLP pipeline against a random sample of notes without requiring keyword selection to perform additional validations. Additionally, our study provides a baseline framework for identifying discussions of prediabetes. Next steps could apply NLP pipelines to identify when discussions about prediabetes meet the threshold for delivery of guideline-concordant care.

### Conclusion

Our NLP pipeline successfully identified prediabetes discussions in unstructured notes with precision approximating human annotation. This approach can be used to evaluate prediabetes counseling during patient visits and describe prediabetes management in primary care. Gathering these data is a critical step to inform interventions to improve the delivery of evidence-based prediabetes care to reduce the incidence of type 2 diabetes.

## Appendix

Multimedia Appendix 1 Supplementary methods and tables.

## Abbreviations

CNN: convolutional neural network
EHR: electronic health record
HbA_1c_: hemoglobin A_1c_
ICD: International Classification of Diseases
IRR: interrater reliability
JHU: Johns Hopkins University
ML: machine learning
NB: Gaussian naïve bayes
NLP: natural language processing
NLTK: Natural Language Toolkit
PCP: primary care provider
PMAP: Precision Medicine Analytics Platform
SGD: stochastic gradient descent
SVM: support vector machines
TF-IDF: term frequency-inverse document frequency
