# Supplementary material for: Identification of Prediabetes Discussions in Unstructured Clinical Documentation: Validation of a Natural Language Processing Algorithm
Source: JMIR Med Inform. 2022 Feb 24;10(2):e29803. doi: 10.2196/29803 (PMC8914791; doi:10.2196/29803)
Supplement: Multimedia Appendix 1 [file medinform_v10i2e29803_app1.docx]

**Supplementary Files**

**Appendix Methods**

**Machine learning classification**

***Conventional Binary Classification Models***

Logistic regression and SVMs are discriminative linear classifiers [1,2]; discriminative training optimizes the algorithm to limit discrepancies between the classifier’s output and the training set responses while applying regularization to prevent overfitting [1]. SGD generates a predictive function using an iterative algorithm to estimate a minimum slope from a subset of the data [3,4]. Decision trees apply simple “if-then-else” decision rules to support class labels, thereby creating a predictive model [5]. Random forests expand on this method by overlaying randomized decision trees and averaging the predictions to decrease overfitting [6]. Finally, NB is a probabilistic classifier, categorizing text from the training set by applying Bayes’ theorem [7].

**Table S1.** Keywords included in search strategy for "prediabetes" concept extraction and frequency of keyword matches in clinical notes (N=519). There were 930 total keyword matches. Results are further stratified by whether the keyword match identified a clinical discussion about prediabetes. Results are presented as N (%).

|  | **All keyword matches** | | **Keyword match identified clinical discussion of prediabetes between patient and provider?** | | | |
| --- | --- | --- | --- | --- | --- | --- |
| **Keyword** | **N=930** | | **Yes (N=641)** | | **No (N=289)** | |
| Prediabetes | 450 | (48) | 297 | (46) | 153 | (53) |
| Impaired fasting glucose | 131 | (14) | 86 | (13) | 45 | (16) |
| Hyperglycemia | 128 | (14) | 116 | (18) | 12 | (4) |
| Pre-diabetes | 86 | (9) | 50 | (8) | 36 | (12) |
| Impaired glucose tolerance | 41 | (4) | 22 | (3) | 19 | (7) |
| Elevated hemoglobin A1c | 23 | (2) | 19 | (3) | 4 | (1) |
| Pre-DM | 20 | (2) | 14 | (2) | 6 | (2) |
| Elevated glucose | 17 | (2) | 12 | (2) | 5 | (2) |
| Glucose intolerance | 11 | (1) | 7 | (1) | 4 | (1) |
| Elevated fasting glucose | 9 | (1) | 9 | (1) | 0 | (0) |
| Borderline diabetes | 6 | (0.7) | 2 | (0.3) | 4 | (1) |
| Elevated A1c | 6 | (0.7) | 6 | (1) | 0 | (0) |
| Increased risk of diabetes | 1 | (0.1) | 0 | (0) | 1 | (0.4) |
| PreDM | 1 | (0.1) | 1 | (0.2) | 0 | (0) |
| Elevated diabetes risk^a^ | 0 | (0) | 0 | (0) | 0 | (0) |
| Increased diabetes risk^a^ | 0 | (0) | 0 | (0) | 0 | (0) |
| Pre DM^a^ | 0 | (0) | 0 | (0) | 0 | (0) |
| Dysglycemia^a^ | 0 | (0) | 0 | (0) | 0 | (0) |
| Pre diabetes^a^ | 0 | (0) | 0 | (0) | 0 | (0) |
| ^a^These keywords were included in keyword search but did not match to any notes. | | | | |  |  |

**Table S2.** spaCy EntityRuler patterns to describe prediabetes discussions. Nouns and verbs are in lemma form to account for morphological variations. These patterns were used to test a Rule-Based Classification system to identify prediabetes discussions.

| **Patterns for Rule-Based Classification** | |  |
| --- | --- | --- |
| present for prediabetes | risk * develop diabetes | weight loss goal |
| discuss prediabetes | recheck * a1c | discuss metformin |
| discuss risk * diabetes | recheck hemoglobin a1c | benefit * metformin |
| new prediabetes diagnosis | lifestyle change | diabetes prevention program |
| diagnose with prediabetes | lifestyle modification | DPP |
| new hyperglycemia | cut * sugar | lifestyle program |
| lab * hyperglycemia | limit sugar | nutrition referral |
| impair fast glucose * lab | sugar sweetened beverage | review risk * * diabetes |
| history * hyperglycemia | eliminate soda | prediabetes * discuss |
| last hemoglobin A1c elevate | limit carbohydrate * diet | impaired fasting glucose* discuss |
| last * a1c elevate | diet modification | hyperglycemia * discuss |
| increase * a1c | recommend exercise | prediabetes * recommend |
| elevate glucose * fast lab | increase physical activity | impaired fasting glucose* recommend |
| review * lab * for prediabetes | recommend weight loss | hyperglycemia* recommend |
| risk * diabetes |  |  |

**References**

1. Ng AY, Jordan MI. On Discriminative versus Generative Classifiers: A Comparison of Logistic Regression and Naive Bayes. Adv. Neural Inform. Process. Syst 2001;14:605-10. <https://ai.stanford.edu/~ang/papers/nips01-discriminativegenerative.pdf>.
2. scikit-learn. 1.1. Linear Models. Secondary 1.1.11 Logistic regression 2019. <https://scikit-learn.org/stable/modules/linear_model.html#logistic-regression> (accessed Jun 2020).
3. scikit-learn. 1.5. Stochastic Gradient Descent. 2019. <https://scikit-learn.org/stable/modules/sgd.html> (accessed Jun 2020).
4. Bottou L, Bousquet O. The Tradeoffs of Large Scale Learning. In Sra S, Nowozin S, Wright SJ (eds). Optimization for Machine Learning. Cambridge: MIT Press, 2012. <https://proceedings.neurips.cc/paper/2007/file/0d3180d672e08b4c5312dcdafdf6ef36-Paper.pdf>.
5. scikit-learn. 1.10. Decision Trees 2019. <https://scikit-learn.org/stable/modules/tree.html> (accessed Jun 2020).
6. scikit-learn. 1.11. Ensemble methods. Secondary 1.11.2. Forests of randomized trees 2019. <https://scikit-learn.org/stable/modules/ensemble.html#forest> (accessed Jun 2020).
7. scikit-learn. 1.9. Naive Bayes. <https://scikit-learn.org/stable/modules/naive_bayes> (accessed Jun 2020).
